# Investigating population continuity with ancient DNA under a spatially explicit simulation framework

**DOI:** 10.1186/s12863-017-0575-6

**Published:** 2017-12-15

**Authors:** Nuno Miguel Silva, Jeremy Rio, Mathias Currat

**Affiliations:** 1 AGP lab, Department of Genetics & Evolution - Anthropology Unit, University of Geneva, Geneva, Switzerland; 2 Institute of Genetics and Genomics in Geneva (IGE3), University of Geneva, Geneva, Switzerland

**Keywords:** Population genetics, Spatially-explicit simulations, Serial coalescent, Ancient DNA, Population continuity, European Neolithic

## Abstract

**Background:**

Recent advances in sequencing technologies have allowed for the retrieval of ancient DNA data (aDNA) from skeletal remains, providing direct genetic snapshots from diverse periods of human prehistory. Comparing samples taken in the same region but at different times, hereafter called “serial samples”, may indicate whether there is continuity in the peopling history of that area or whether an immigration of a genetically different population has occurred between the two sampling times. However, the exploration of genetic relationships between serial samples generally ignores their geographical locations and the spatiotemporal dynamics of populations. Here, we present a new coalescent-based, spatially explicit modelling approach to investigate population continuity using aDNA, which includes two fundamental elements neglected in previous methods: population structure and migration. The approach also considers the extensive temporal and geographical variance that is commonly found in aDNA population samples.

**Results:**

We first showed that our spatially explicit approach is more conservative than the previous (panmictic) approach and should be preferred to test for population continuity, especially when small and isolated populations are considered. We then applied our method to two mitochondrial datasets from Germany and France, both including modern and ancient lineages dating from the early Neolithic. The results clearly reject population continuity for the maternal line over the last 7500 years for the German dataset but not for the French dataset, suggesting regional heterogeneity in post-Neolithic migratory processes.

**Conclusions:**

Here, we demonstrate the benefits of using a spatially explicit method when investigating population continuity with aDNA. It constitutes an improvement over panmictic methods by considering the spatiotemporal dynamics of genetic lineages and the precise location of ancient samples. The method can be used to investigate population continuity between any pair of serial samples (ancient-ancient or ancient-modern) and to investigate more complex evolutionary scenarios. Although we based our study on mitochondrial DNA sequences, diploid molecular markers of different types (DNA, SNP, STR) can also be simulated with our approach. It thus constitutes a promising tool for the analysis of the numerous aDNA datasets being produced, including genome wide data, in humans but also in many other species.

**Electronic supplementary material:**

The online version of this article (10.1186/s12863-017-0575-6) contains supplementary material, which is available to authorized users.

## Background

Genetic diversity in human populations reflects past demographic changes and migrations. While genetic data from contemporary humans have long been the sole source of molecular data used to draw inferences on the evolution and peopling history of their ancestors e.g., [1], direct evidences from the past have been recently recovered by sequencing ancient DNA (aDNA) from different time periods and geographical areas [2–5]. Those data have been commonly used to assess whether two samples from different periods but located in the same geographical area (hereafter called “serial samples”) come from a single population [6–9]. This hypothesis is usually defined as population continuity through time and contrasts with scenarios of large genetic input or replacement due to the arrival of immigrants leading to an increased level of genetic differentiation between the serial samples [2, 10]. In different regions of Europe, population continuity was often rejected when ancient mitochondrial DNA from populations spanning from prehistoric periods until modern days was analysed [11], meaning that the observed shifts in allele frequencies cannot be explained by genetic drift alone and that substantial demographic replacements must be invoked.

To assess whether two genetic samples from different time periods may be considered as coming from a single population evolving under the sole effect of genetic drift, a model-based test has been developed and applied to mitochondrial data [2]. The framework of this test is to simulate serial genetic samples with the same characteristics as the real ones (time period, sample size) issued from a single panmictic population and to compute an index of genetic differentiation between those samples, usually *Fst*. With a reasonable number of simulations (usually several thousands), a distribution of genetic distances between serial samples under the null hypothesis of population continuity is provided. If the observed genetic distance between the real samples is above 95% of the simulated ones, then the null hypothesis is rejected, meaning that genetic drift alone is not able to generate the differences in genetic diversity between the serial samples. Genetic shift through demographic replacement or migration are the factors commonly proposed to explain an absence of population continuity. For instance, a rejection of population continuity between Palaeolithic hunter-gatherers and Neolithic farmers from the same area could be interpreted as a demic replacement of hunter-gatherers by farmers arriving from another region [2]. This test for population continuity thus relies heavily on the simulated distribution of *Fst* between two samples and consequently on the underlying model, which must be as realistic as possible.

However, while being able to simulate complex scenarios and some very simple levels of population structure, coalescent-based models developed so far to test the hypothesis of population continuity with aDNA do not consider ancestral migration of genes among neighbouring populations in a spatially explicit context [12–14]. They thus make the strong assumption that the ancestral lineages of people living today at a given place have always been in the same area in the past [15]. This assumption of panmixia, or near panmixia, through time is questionable given human mobility. Indeed, studies at regional levels have highlighted the major role played by migrations in partially renewing the local genetic pool over a short period [16–19]. The rare studies incorporating ancient population structure when analysing aDNA, although in a simplified way, better explained the data [4, 20, 21]. Moreover, the incorporation of a spatial component in the analysis of genetic data has already generated insights into human evolution and even some counter-intuitive results that were undetectable with panmictic approaches [22–25].

Here, we explore the effects of incorporating population structure and migration as fundamental elements when investigating the genetic relationships between serial samples. We used a modified version of the program SPLATCHE2 [26], allowing for the sampling of genetic lineages at a given time and location. Therefore, we improve the test for population continuity by incorporating the effects of regional gene flow. Indeed, when testing for population continuity using aDNA, the effect of migration over time within a structured population should be considered to avoid confounding its impacts with a genetic shift due to complete or partial demographic replacement through a large immigration wave. Furthermore, we also investigate how the temporal and spatial variation in sampling that usually characterize aDNA population samples (due to the scarcity of fossils and DNA preservation issues) may affect population continuity analyses. Our spatially explicit approach copes with these two dimensions simultaneously, while only temporal heterogeneity within population samples can easily be considered in the programs used so far to test for population continuity, such as BSSC [12] and FastSimCoal [13]. We apply our simulation approach to two mitochondrial European datasets, one in Germany and one in France, each comprising an ancient sample from the early Neolithic period and a modern sample. We test population continuity in these two areas using panmictic and spatial coalescent approaches and compare the results.

## Methods

### Spatially explicit simulation of ancient DNA

**Fig. 1:**
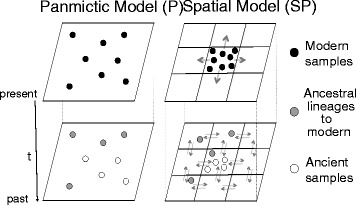
Schematic representation of lineages simulated under panmictic (P) and spatial models (SP) of population continuity. The P representation stresses the geographical constraint imposed, with individuals staying in the same location during the whole simulation. This constraint is absent in SP, with migration and population structure adding an important realistic element

A modified version of the program SPLATCHE2 [26] allowing for the sampling of lineages at different time points was used to perform spatially explicit simulations of ancient DNA. This version (available at www.splatche.com) improves the model-based coalescent approaches previously used to test for population continuity with aDNA data [12, 13] by explicitly considering the spatial dynamics of genes through time (Fig. 1). The framework comprises, as a first step, the simulation of a population expansion in a grid of demes exchanging migrants in a stepping-stone fashion. In a second step, a serial coalescent reconstruction is performed to generate genetic diversity in samples of different ages and locations, drawn from the simulated population. The genealogy of simulated lineages is reconstructed conditional on the density and migration values calculated during the first step [27]. The genetic diversity of those lineages is simulated by distributing mutations on the coalescent tree. See the original description of SPLATCHE2 for more details on the algorithms [26].

We used two spatially explicit frameworks for performing simulations:

(1) a virtual square map in which we explored the influence of the spatial components when investigating the relationship between serial samples and other genetic diversity statistics;

(2) a realistic European map, which was used to apply our approach to two real datasets to test for population continuity.

### Simulations in a virtual square map

#### Effects of local gene flow on genetic distance among serial samples

We first explored the effects of incorporating population structure and migration when investigating the relationship between two population samples from the same area but from different periods. The integration of those factors could have important consequences when testing for population continuity, as the commonly used framework heavily relies on the simulated distribution of genetic distances, to which real data are compared, and consequently on the underlying model, which must be as realistic as possible.

To represent population continuity through time, we thus simulated the expansion of one population in a grid of demes exchanging migrants (spatial model, SP) or a simple demographic growth in a single deme (panmictic model, P). For SP, we simulated the growth of a population of 100 haploid individuals during 2000 generations (~50,000 years for humans) from the centre of a square map of 2500 demes (50 × 50), with a carrying capacity *K* of 500 haploid individuals each, following the method developed in [25]. We used a generation time of 25 years, which is appropriate for representing female generation intervals [28] and thus for the study of mitochondrial DNA. A logistic equation controls the population growth at the deme level with a growth rate *r*. This spatial model, SP, was compared to a panmictic version, P, which also simulated the growth of a population of 100 haploid individuals during 2000 generations. The P model was made up of only one deme with parameters adapted for the purpose of comparison with the spatial model: the P model included no migration (*m* = 0), the population growth was equal to 0.012 to reach *K*_*end*_ at the end of the simulation, with *K*_*end*_ = D*K, where *D* is the number of demes and *K* the carrying capacity in SP models (*K*_*end*_ = 500 individuals × 2500 demes). Note that all parameters are constant through the whole simulation unless otherwise indicated.

For SP, we performed several series of simulations with various migration rates *m* (0.005, 0.01, 0.05 and 0.1) to explore the effect of population structure on the genetic differentiation between two serial samples drawn from a continuous population. *Nm* is the migration rate *m* multiplied by the equilibrium size *K* of the deme, and defines the number of migrants *Nm* that are distributed in the demes located adjacently at each generation (at a maximum of four neighbours: North, South, East and West). We use the term *Nm* instead of *Km* by convention with other articles [25]. A high *Nm* means that the population is large and interconnected, while a small *Nm* means that the population is divided in small and isolated units. For all scenarios, *Nm* is kept constant throughout the simulation except for the “Neolithic” scenario, where *Nm* was changed from 5 to 50, 400 generations before present, to reflect changes following the Neolithic transition [29]. This was done by modifying *m* in all demes at the same time. We also investigated the effect of various combinations of *K* (100, 150 and 200) and *m* resulting in an identical *Nm* of 5 or 50.

Because relatively large datasets belonging to the same prehistoric “population”, defined either geographically or culturally, have mostly been published for mitochondrial HVS1, such as for the late Upper Paleolithic and Neolithic era in Central and Western Europe [2, 4, 30], we simulated sequences representing mtDNA. However, SPLATCHE2 is able to simulate many different kinds of molecular data, haploid or diploid, such as DNA sequences, SNP, STR, RFLP, including recombination between loci. The approach presented here is thus also suited to study genome wide aDNA in a spatial context. For each simulation, two groups of 30 mtDNA lineages, called “population samples” hereafter, were sampled in the centre of the map at two different times: a modern population sample at present and an ancient population sample 400 generations before the present), which corresponds approximately to the beginning of the Neolithic transition (~10,000 BP). A mutation rate *μ* = 3.3 × 10^–6^ for a DNA sequence of 300 bp was simulated to approximate mtDNA diversity in European populations [25].

A measure of genetic differentiation computed as *Fst* between the modern and ancient samples, as well as the gene diversity within each sample (modern *H*_*mod*_ or ancient *H*_*anc*_) and the nucleotide diversity within each sample (modern *π*_*mod*_ or ancient *π*_*anc*_), were computed using the software Arlequin 3.5 [31]. We also computed the average coalescent time $\overline{t_1}$ between the ancient and modern population samples as well as the average coalescent time $\overline{t_0}$ within population samples ($\overline{t_0}$_*anc*_ and $\overline{t_0}$_*mod*_ within only the ancient or only the modern population samples, respectively). To better explore the stochastic nature of the coalescent process, 10,000 independent simulations were performed for each scenario (defined as a combination of parameters). The final results are presented as a distribution of *Fst* for each scenario, which could be used as a null distribution of genetic differentiation under the hypothesis of population continuity following the procedure described in [2].

#### Comparison between panmictic and spatial models when testing population continuity

To illustrate the difference between using SP or P models for testing population continuity, we simulated four sets of 1000 pseudo-observed datasets (pods) under four different continuity scenarios (P, SP-*Nm* = 50, SP-*Nm* = 25 and SP-*Nm* = 5). For each set, we then computed the probability of rejecting continuity with each of the four scenarios. The *Fst* of each pod is computed and compared to the distribution of *Fst* computed from 1000 simulations generated under each scenario. If the proportion of simulated *Fst* bigger than the pod’s *Fst* is below a 5% threshold, then the null hypothesis of population continuity is rejected for that pod under the scenario that generated the distribution. For each scenario, we repeated this procedure for the whole set of 1000 pods and computed the proportion of pods which reject continuity at the 5% level. This represents a measure of type 1 error - i.e., the probability of rejecting the null hypothesis of continuity when it is true - because all scenarios simulate continuity with more (SP-*Nm* = 5) or less (P) population structure.

#### Spatial and temporal variance within ancient population samples

Because of the scarcity of fossils and DNA preservation issues, lineages quite distant in time and space are often grouped together based on cultural or geographical criteria, which constitutes one of the main differences with modern samples. Thus, in two other series of simulations in the square map, we explored the effects of spatial and temporal heterogeneity between aDNA sequences belonging to the same ancient population sample on the comparison between serial samples.

**Fig. 2:**
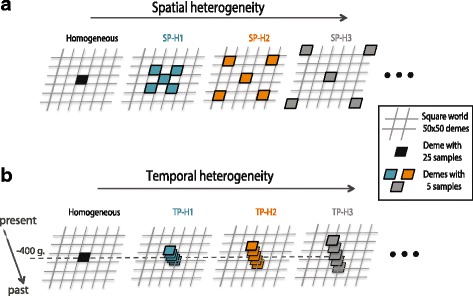
Simulation of spatial and temporal variance in the ancient population sample. Modern lineages are always taken in one single central deme (black square). **a** Ancient lineages are drawn in groups of 5 in 5 different demes, separated geographically with increasing distance from the centre, along the two diagonals, SP-H1 is the least dispersed and SP-H7 the most dispersed. **b** The same principle is applied for the temporal variation in ancient sampling, with TP-H1 being the least variable and TP-H4 the most. A homogenous sampling (time and location) is shown for comparison (all 25 ancient lineages taken in the black square)

We varied the heterogeneity within the ancient dataset for two different levels of population structure reflected by *Nm* values of 5 or 50, dividing the 25 ancient lineages into five groups as shown in Fig. 2 and described below. Although the ancient dataset was divided into five groups of lineages located in different demes, it was considered as a single ancient population sample when computing *Fst* with the modern sample to reflect the kind of grouping usually made with aDNA [2, 32].

For investigating spatial heterogeneity (Fig. 2a), we tested seven different spatial configurations, all with one group of five lineages in the deme located in the centre of the map and four other groups of five lineages each taken in four demes along the diagonals of the square area and with increasing distances from the centre deme (sampling “SP-H1” to “SP-H7”). For investigating temporal heterogeneity (Fig. 2b), we tested four temporal configurations, with all lineages taken in the central deme but at different times. One group of five lineages is always drawn 400 generations before the present, but two other groups of five are taken more recently, and the two others at more ancient times. The temporal variance increases from the “TP-H1” to the “TP-H4” scenarios. This heterogeneous sampling scheme was compared to a homogeneous sampling scheme for which all 25 lineages were taken from the central deme 400 generations before the end of the simulation.

### Application to real data in Europe

#### Spatial Scenario - SP

We then implemented our approach on a digital map of Europe, divided into roughly 7000 inland demes of 50 × 50 *km* (Additional file 1 Figure S1), to test for population continuity in Germany and France. We incorporated into our spatial framework the two phases of growth model used to test for population continuity in [2], thus simulating continuity since the initial colonization of Europe approximately 40,000 years ago. We used the same map and parameters as in [33] to simulate the evolution of European populations from the arrival of modern humans until today, including the Neolithic transition. In a few words, a population starting with 100 haploid individuals expands during 1600 generations from a deme located in the Middle East, with *r* = 0.4 and *m* = 0.25, until the whole map is occupied. The Neolithic transition is represented by an instantaneous demographic increase in all demes at the same time, the *K* of each deme changing from 40 to 250 effective genes after 1200 generations, a date that corresponds approximately to the beginning of the Neolithic transition [34]. Compared to the original model, the *K* of Neolithic populations was updated to a more realistic value (250 instead of 800) corresponding to the estimated density for Neolithic populations in central Europe (~0.6 individuals/*km* ^*2*^,) [29]. However, we also tested larger (*K* = 500) and lower (*K* = 100) values (Additional file 2 Table S1) to explore *Nm* in the range (25–125) estimated for post-Neolithic populations [35]. Note that *K* is given in effective size for mitochondrial data (autosomal size/4) and that the number of emigrant *Nm* is spread over all neighbouring demes. In order to represent a progressive population increase during historical times and reach an effective female population size of about 12 million in central Europe at the end of the simulation, as in [2], we changed *K* to 24,000 at generation 1520 (~2000 years before present).

We also implemented a one phase of growth model simulating a full replacement of European hunter-gatherers by farmers from Anatolia during the Neolithic e.g. [36, 37]. This scenario thus simulates population continuity for the last 10,000 years in Europe and was implemented with parameters from [33] and the same Neolithic *K* values than for the two phases of growth model described above (Additional file 2 Table S1).

#### Panmictic scenario – P

A non-spatial scenario was also used for the purpose of comparison with the spatially explicit model. This scenario P is similar to SP but in a single deme, without any spatial component or migratory factor.

A population of 100 haploid individuals grows exponentially with a rate *r =* 0.016 during 1600 generations, and *K* changes from 20,000 (*K*_*P*_) to 12,000,000 (*K*_*M*_) after 1200 generations. The values of *K*_*P*_ and *K*_*M*_ are equal to the sum of carrying capacities for all demes from central Europe (500 demes) at the corresponding period in the spatial map (compare scenarios P2-k20 and SP2-k250 in Additional file 2 Table S1). To check the robustness of the results, we explored larger intermediate *K* (Neolithic) values in the range used in [2] (50,000 and 100,000), with the growth rate modified to reach the corresponding *K* by the end of each period (Additional file 2 Table S1). As for SP, we also simulated a model of one phase of exponential growth during 400 generations, representing population continuity from the Neolithic era to the present (Additional file 2 Table S1).

#### Genetic data simulated

To assess population continuity in Germany and France, we reproduced by simulation mitochondrial samples identical to real data in terms of lineage number, location and age for the spatial scenario (SP) but considering only their age and number for the panmictic scenario (P). We chose these two areas because relatively large ancient population samples have been published for both (Table 1). For Germany, we used a Neolithic population sample of 23 lineages (we excluded 2 lineages from the original dataset [2] based on their geographical remoteness) and 50 modern Germans [38]. The French dataset includes 39 Neolithic lineages [39] and 50 modern French [40]. After the exclusion of the positions with missing data, we simulated mitochondrial HV1 DNA sequences of 341 bp (Germany) and 358 bp (France) using a mutation rate of 7.9 × 10^–6^ mutations/generation/site [41].

**Table 1:** Mitochondrial datasets used to test for population continuity in Germany and France

*geographic region*	*group*	*sample count (group)*	*site*	*sample age (cal BC)*	*latitude*	*longitude*	*individuals per site*	*reference*
Germany	Modern	50		Present	51.87	10.91	50	[38]
Germany	Early Neolithic (LBK)	23	Derenburg	5500–4775	51.87	10.91	5	[2]
			Halberstadt-Sonntagsfeld	5500–4775	51.90	11.06	3	
			Oberwiederstedt 1, Unterwiederstedt	5500–4775	51.67	11.53	2	
			Eilsleben	5000	52.15	11.22	1	
			Schwetzingen	5500–4775	49.38	8.57	4	
			Vaihingen	5500–4775	48.93	8.96	1	
			Unseburg	5500–4775	51.93	11.52	1	
			Seehausen	5500–4775	51.33	11.13	1	
			Flomborn	5500–4775	49.69	8.15	5	
France	Modern	50		Present	48.70	2.45	50	[40]
France	Early Neolithic	39	Gurgy	5750	47.95	3.54	39	[39]

## Results

### Virtual square map: Effects of local gene flow on the genetic distance between serial samples

First, we investigated the effects of population structure and migration when computing the genetic distance between an ancient population sample taken approximately 10,000 years ago (representing the beginning of the Neolithic period) and a modern population sample taken at present. We simulated the evolution of genetic diversity in a continuous population either for a panmictic scenario (P) representing a single population or for a series of spatial scenarios (SP) considering a structured population with various intensities of genetic exchange between neighbouring demes, smaller *Nm* meaning less absolute gene flow between demes.

**Fig. 3:**
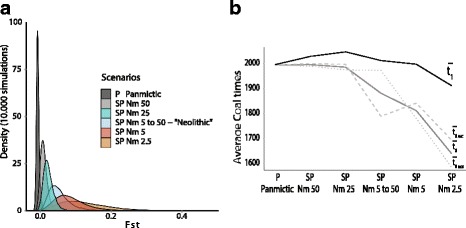
Effects of population structure on the genetic differentiation (*Fst*) between serial samples. From a panmictic scenario P (one single deme) to spatial scenarios (SP) with increasing levels of population structure (inversely proportional to *Nm*) among 2500 interconnected demes. **a** *Fst* distributions and (**b**) Average coalescent times at the intra-population ($\overline{t_0}$) and inter-population levels ($\overline{t_1}$)

**Fig. 4:**
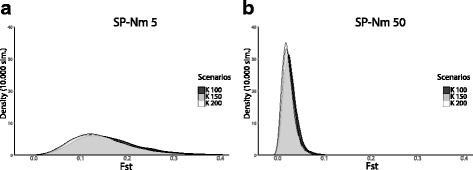
*Fst* distributions between modern and ancient population samples calculated for different combinations of *K* (100,150 and 200) and *m* resulting either in *Nm* 5 (**a**) or *Nm* 50 (**b**)

In comparison to P, all SP scenarios give larger *Fst* means and variances between the two simulated serial samples (Fig. 3a and Table 2). Among the SP scenarios, *Fst* and its variance tend to increase when *Nm* decreases. For a given *Nm* value (5 or 50), there is only slight differences among *Fst* distributions (Fig. 4), smaller population sizes (smaller *K*) lead to slightly increased *Fst* and decreased genetic diversity. All statistics are more affected when the structure is strong (*Nm* 5) rather than weak (*Nm* 50). The “Neolithic” scenario simulates a shift from *Nm* 5 to *Nm* 50, 400 generations before the present (~10,000 years ago) to represent the demographic changes that accompanied this profound economic and cultural transition. The “Neolithic” *Fst* distribution was found to be between those for *Nm* 5 and *Nm* 50 but slightly closer to *Nm 5*, which indicates that the demographic dynamics through time affect the simulated genetic diversity and that gene flow during the Paleolithic time has more influence on current genetic diversity than during the Neolithic time (Table 2).

**Table 2:** Parameters and statistics for the various scenarios simulated in the square world. P = panmictic model, SP = spatially-explicit models

*Model*	*Nm*	*K*	*m*	*FST*_*mod* vs *anc*_	*H*_*mod*_	*H*_*anc*_	*π*_*mod*_	*π*_*anc*_	$\overline{t_1}$	$\overline{t_0}$_*mod*_	$\overline{t_0}$_*anc*_	$\overline{t_0}$
P	*–*	*1′250’000*	*0*	*0.001 ± 0.006*	*0.98 ± 0.02*	*0.96 ± 0.04*	*3.93 ± 0.62*	*3.15 ± 0.58*	*1985 ± 1*	*1985 ± 1*	*1985 ± 1*	*1985 ± 000*
SP	*50*	*500*	*0.1*	*0.021 ± 0.013*	*0.96 ± 0.03*	*0.94 ± 0.04*	*3.91 ± 0.72*	*3.17 ± 0.67*	*2024 ± 2*	*1981 ± 3*	*1992 ± 3*	*1987 ± 008*
	*50*	*200*	*0.25*	*0.024 ± 0.014*	*0.96 ± 0.03*	*0.94 ± 0.04*	*3.91 ± 0.73*	*3.16 ± 0.68*	*2025 ± 2*	*1976 ± 4*	*1987 ± 3*	*1981 ± 008*
	*50*	*150*	*0.33*	*0.026 ± 0.015*	*0.95 ± 0.03*	*0.94 ± 0.04*	*3.90 ± 0.75*	*3.15 ± 0.69*	*2022 ± 2*	*1967 ± 4*	*1983 ± 3*	*1975 ± 011*
	*50*	*100*	*0.5*	*0.029 ± 0.015*	*0.95 ± 0.03*	*0.93 ± 0.04*	*3.89 ± 0.76*	*3.14 ± 0.69*	*2019 ± 2*	*1959 ± 4*	*1974 ± 3*	*1966 ± 011*
	*25*	*500*	*0.05*	*0.036 ± 0.018*	*0.95 ± 0.03*	*0.93 ± 0.05*	*3.89 ± 0.77*	*3.14 ± 0.69*	*2042 ± 2*	*1964 ± 4*	*1988 ± 3*	*1976 ± 017*
	*5 to 50*	*500*	*0.01–0.1*	*0.075 ± 0.041*	*0.96 ± 0.03*	*0.84 ± 0.08*	*3.92 ± 0.73*	*2.76 ± 0.88*	*2015 ± 2*	*1962 ± 4*	*1761 ± 6*	*1862 ± 142*
	*5*	*500*	*0.01*	*0.113 ± 0.058*	*0.86 ± 0.07*	*0.84 ± 0.08*	*3.46 ± 0.97*	*2.82 ± 0.88*	*1992 ± 3*	*1752 ± 7*	*1819 ± 6*	*1786 ± 047*
	*5*	*200*	*0.025*	*0.137 ± 0.065*	*0.83 ± 0.08*	*0.82 ± 0.09*	*3.36 ± 1.00*	*2.77 ± 0.90*	*1999 ± 3*	*1678 ± 8*	*1762 ± 6*	*1720 ± 059*
	*5*	*150*	*0.033*	*0.163 ± 0.077*	*0.80 ± 0.09*	*0.80 ± 0.09*	*3.25 ± 1.06*	*2.68 ± 0.93*	*1995 ± 3*	*1665 ± 7*	*1757 ± 6*	*1711 ± 064*
	*5*	*100*	*0.05*	*0.168 ± 0.077*	*0.81 ± 0.09*	*0.79 ± 0.10*	*3.26 ± 1.03*	*2.62 ± 0.92*	*2001 ± 3*	*1656 ± 8*	*1732 ± 7*	*1694 ± 054*
	*2.5*	*500*	*0.005*	*0.162 ± 0.093*	*0.79 ± 0.10*	*0.78 ± 0.11*	*3.04 ± 1.07*	*2.54 ± 0.93*	*1886 ± 5*	*1538 ± 8*	*1657 ± 7*	*1598 ± 084*

Table 2 shows that intra-population diversity statistics measured in both ancient and modern samples (gene diversity *H* and nucleotide diversity *π*) decrease with population structure. Higher values for both statistics are found for scenario P while lower values are found for the SP scenario with *Nm* 2.5. The difference between scenarios is greater for *H* than for *π*.

For the panmictic scenario P, $\overline{t_0}$ and $\overline{t_1}$ are similar because most of the coalescence occurs close to the root of the tree, at the onset of the demographic expansion when the population size is small with modern and ancient lineages sharing common ancestors. For spatial scenarios, a decreasing *Nm* favours earlier coalescent events between lineages from the same population sample, as shown by diminishing $\overline{t_0}.\mathrm{Note}\  \mathrm{that}\ \overline{t_0}$_*mod*_ is slightly more affected than $\overline{t_0}$_*anc*_, and that $\overline{t_1}$ also diminishes but less than $\overline{t_0}$ (Fig. 3b). Increasing the population structure thus results in smaller genetic diversity within population samples (*π*_*mod*_ *, π*_*anc,*_ *H*_*mod*_ and *H*_*anc*_ in Table 2) and in more genetic differentiation between serial population samples, as measured by the *Fst*, which is proportional to $\frac{{\overline{t}}_1-{\overline{t}}_0}{{\overline{t}}_1}$ [42]. The particular cases of identical *Nm* with varying *K* show that the decrease in $\overline{t_0}$ is more pronounced than the decrease in $\overline{t_1}$, especially when *Nm* is small (Table 2).

### Virtual square map: Comparison between panmictic and spatial models when testing population continuity

Table 3 shows the rate of rejection of the population continuity hypothesis when tested with four different scenarios simulating population continuity with various levels of population structure (from full panmixia, P, to strong spatial structure, SP-*Nm5*). 1000 pseudo observed datasets (pods) have been generated with each of the four scenarios (columns) and their *Fst* have been compared to the *Fst* distribution generated by 1000 simulations under each of the four scenarios (lines). Because all scenarios simulate continuity, a rejection thus corresponds to a type 1 error (rejection of a true null hypothesis). As expected, the rate of type 1 error is equal to the chosen threshold (5%) when pods are tested with the scenario that generated them (diagonal). We find that for all spatial scenario, the probability of type I error when testing for population continuity with the P model is much higher than with any SP scenario. It shows that the spatial model is more conservative than the panmictic model when testing population continuity. More generally, when pods generated under a scenario with a given population structure are tested with scenarios simulating less structure, the rate of type 1 error is high (upper triangle of Table 3), while it is always very low when they are tested with more structure (lower triangle of Table 3).

**Table 3:** Rates of type I error measured by generating 1000 pseudo-observed datasets with a known continuity scenario and testing for population continuity with four sets of 1000 simulations generated under four different continuity scenarios (P = panmictic, SP = spatially-explicit with various intensities of gene flow, from the lowest *Nm*5 to the highest *Nm*50)

		*Continuity scenarios used to generate pseudo-observed data*			
		*P*	*SP Nm50*	*SP Nm25*	*SP Nm5*
*Simulated continuity scenarios*	*P*	0.05	0.81	0.96	1.00
	*SP Nm50*	0.00	0.05	0.27	0.93
	*SP Nm25*	0.00	0.01	0.05	0.78
	*SP Nm5*	0.00	0.00	0.00	0.05

### Virtual square map: Effects of temporal and spatial variance within ancient samples

**Fig. 5:**
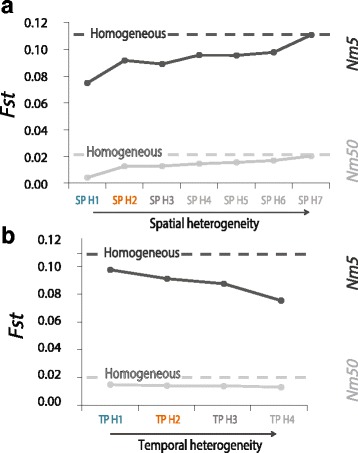
Effects of spatial and temporal variance among lineages within ancient population samples when computing genetic distances between ancient and modern population samples. **a** *Fst* is calculated between the group of modern lineages and the group of ancient lineages considered as one population sample. *Fst* values are given for all sampling schemes in comparison with a homogeneous sampling (dotted horizontal line), with SP-H1 being the less heterogeneous and SP-H7 being the most diverse, for *Nm* 5 (dark grey) and *Nm* 50 (light grey). **b** The same principle is applied for the variation in temporal sampling, with TP-H1 being the least variable in terms of ages and the TP-H4 the most. See Fig. 2 for a graphical representation of the sampling schemes

To explore how spatial and temporal heterogeneity within the ancient population sample affect its genetic relationship with a modern sample (taken in one single deme), we simulated again a continuous and structured population. We used either a small-structured population representing Paleolithic hunter-gatherers (*Nm* 5) or a larger population with more gene flow representing late Neolithic farmers or historical populations (*Nm* 50). Then, we computed the *Fst* between the ancient and the modern population samples. Our results show that compared to a homogeneous sampling in one single location and time, *Fst* tend to be smaller with heterogeneity within the ancient sample. More specifically, *Fst* tends to increase with spatial heterogeneity and reaches a value equivalent to a homogenous sampling when heterogeneity is big enough (see SP-H7 in Fig. 5a). In contrast, *Fst* tends to decrease with temporal heterogeneity (Fig. 5b). Both trends are stronger when *Nm* is smaller. Overall, these results show that the combined effect of the variations in time and location of lineages within the ancient population sample do influence its genetic relationship with another serial sample in a way that is difficult to predict. These parameters should thus be taken into account, especially when the population under study is small and isolated.

### Realistic European map: Testing population continuity using our spatially explicit approach

**Fig 6:**
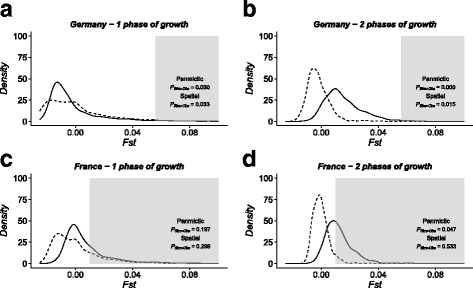
*Fst* distributions simulated between early Neolithic and modern samples in Germany (**a**-**b**) and in France (**c**-**d**), under different population continuity models. Distributions for a panmictic model P (dashed line) and spatially explicit model SP (solid line) are shown for a one phase of growth scenario (**a** and **c**, continuity during ~10,000 years) and a two phases of growth scenario (**b** and **d**, continuity during ~40,000 years). The grey area represents the values bigger than the observed *Fst* estimated on the real data. The proportion of simulated values greater than the observed values (*P*_*sim > obs*_) is given for both models

The *Fst* between the two serial German samples and the two serial French samples are estimated to 0.056 (*p-value* = 0.002) and 0.009 (*p-value* = 0.084), respectively. Our results show that the relationship between Neolithic farmers and modern lineages from Germany cannot be explained by population continuity. Both spatial (SP) and non-spatial (P) models significantly reject population continuity for the one (*P*_*sim > obs*_ ≤ 0.033, Fig. 6a) and two (*P*_*sim > obs*_ ≤ 0.015, Fig. 6b) phases of growth models. Regarding the French dataset, the two phases of growth model rejects the population continuity hypothesis when using the non-spatial model P (*P*_*sim > obs*_ = 0.047) but not when using the spatially explicit one, SP (*P*_*sim > obs*_ = 0.533, Fig. 6d). Still for the French dataset, the one phase of growth model retains the null hypothesis of continuity in both cases (*P*_*sim > obs*_ ≥ 0.197, Fig. 6c). In accordance with the descriptive results obtained in the square world, *P*_*sim > obs*_ is always larger for SP than for the most comparable P scenario (Fig. 6 and grey background in Additional file 3 Table S2). The exploration of parameters (Additional file 3 Table S2) shows that population continuity in Germany is rejected by all scenarios (12/12, 8/12 after Bonferroni correction for multiple tests). Regarding the French dataset, population continuity is never rejected with SP scenarios (0/6) while it is often rejected with P scenarios (4/6, 2/6 after Bonferroni correction for multiple tests).

## Discussion

### Effects of population structure and migration when analysing ancient DNA

We present an original spatially explicit approach that allows for the simulation of genetic diversity in a series of population samples taken at different locations and at different times. Overall, our results underline the need to consider the spatial dynamics of genes when analysing ancient population samples because the effects of the various temporal and spatial processes in action (geography, migration, variance in sampling) are complex and may have contrasting effects. When compared to a panmictic model, we show that models including population structure and migration between neighbouring demes show a rise in the genetic differentiation between serial samples taken at the same location, as measured by the statistic *Fst*. The genetic differentiation between serial samples increases inversely to the composite parameter *Nm*, which represents the amount of gene flow between subdivisions of a structured population (Fig. 3a).

The increase of *Fst* together with the reduction of *Nm* is due to a larger number of coalescent events between lineages belonging to the same population sample (either ancient or modern, $\overline{t}$_*0*_ in Fig. 3b), while the average number of coalescent events between lineages belonging to different samples are much less affected ($\overline{t}$_*1*_ in Fig. 3b). Two effects occurring during the “scattering phase” [43, 44] are involved in the decreases in $\overline{t}$_*0*_. First, a lower *m* decreases the probability of emigrating; thus, lineages tend to stay longer in the deme where they have been initially sampled and are more likely to undergo a coalescence. Second, a lower *N* increases the probability of a coalescence between two lineages located in the same deme. If lineages belonging to the same sample share more coalescences (lower $\overline{t}$_*0*_), they will tend to be more similar to each other and more different from lineages from other samples. The effect of *Nm* on $\overline{t}$_*1*_ is much smaller because, going backward in time, as soon as lineages leave the initial deme(s), they have a very low probability of encountering another lineage due to the large number of demes in the grid, whatever the value of *Nm*. Most of the remaining coalescent events occur close to the onset of the expansion, when the spatial distribution and size of the population are low.

### Heterogeneity within ancient samples

Ancient population samples are often characterized by an extensive temporal and spatial heterogeneity among ancient lineages that are grouped together on the base of cultural or geographical criteria. For instance, the hunter-gatherer sample published in [2] encompasses ancient lineages with a range of dates from 2250 calBCE to 13,400 calBCE and a geographical range including lineages as distant as 3000 *km*. Here, we show that taking into account the geographical and temporal variance within ancient population samples also affects in different ways their genetic relationships with other serial samples. While *Fst* tends to decrease with temporal variance, it tends to increase with spatial variance, but it generally stays below the value measured using a homogeneous sampling (Fig. 5), except when lineages are extremely spread out spatially. These results show that not only the variation in time of the lineages belonging to a population sample but also that their geographical variation matters when testing for inter-population genetic relationships between serial samples.

### Spatial parameters are especially important when analysing small populations

The effects of population structure and sample variance are thus additive and, importantly, are stronger in small isolated populations than in large connected populations (Fig. 3a and Fig. 5). To evaluate the effect of population size varying with time, we simulated a “Neolithic” scenario where *Nm* changes from a low value of 5 to a larger value of 50 at a time corresponding roughly to the beginning of the Neolithic transition (~10,000 years ago). This scenario also shows that the oldest (pre- Neolithic) combination of population demography and gene flow (*Nm*) has a stronger effect on the genetic relationship between the two serial samples than the most recent *Nm* (post-Neolithic). Indeed, the distribution of *Fst* is between the distributions for *Nm* = 5 and *Km* = 50 but is closer to the former. This result demonstrates that taking into account spatial structure in the model is especially suited to studying old prehistoric population samples because the farther back in time we go, the smaller and more isolated the populations were e.g., [45]. For instance, the *Nm* estimated in current hunter-gatherer populations is smaller than 10 [35], and a clear difference is visible between a panmictic model and a spatial model with this level of gene flow (Fig. 3a).

### Inclusion of the spatiotemporal dynamics of genes when investigating population continuity

Ancient DNA is commonly used to test whether two genetic samples drawn from the same area at different times are issued from a single continuous population or, in contrast, if some immigration of people from a different area between the two sampling times must be invoked to explain their genetic differences. To measure the genetic differentiation between the two real serial samples, a summary statistic such as *Fst* is calculated. This statistic can then be confronted with a distribution of *Fst* between virtual samples with the same characteristics as the real ones (time period and sample size) simulated under the null hypothesis of population continuity [2]. If the proportion of simulated *Fst* bigger than the observed value is below a 5% threshold, then the null hypothesis of population continuity is rejected, meaning that genetic drift alone is not able to generate the difference between the serial samples. However, the population continuity test used to date is non-spatial in the sense that it simulates a single panmictic deme without considering any population structure or migration, which are known to be important factors in human evolution [25, 46–48].

Here, we extended the population continuity test by incorporating constant and on-going migration within a structured population, which is a different process than a large genetic replacement due to the arrival of an immigrant population and should not lead to a rejection of the hypothesis of continuity. Under our spatial model, the assumption of genetic continuity between two serial samples implies that they descend from the same population and that the genetic differences between them are not only due to genetic drift and sampling (as in panmictic model) but also to gene flow with neighbouring populations. We demonstrate that including population structure affects significantly the test for population continuity because it increases the genetic distance *Fst* obtained under the null hypothesis with which the real data are confronted (Fig. 3a). Testing for population continuity is thus more conservative when the spatial dynamics of genes are considered in the model because a larger observed *Fst* is necessary to reject the null hypothesis of continuity. Using the panmictic model, the simulation of *Fst* values between serial samples is a biased estimation of the true genetic differentiation towards small values (Fig. 3a), leading to a higher type I error rate (Table 3) if the dataset under analysis comes from a structured population, which is realistic in most situations. Consequently, it is possible that some of the continuity rejections that have been detected so far [11] would vanish if migration and population structure were considered in the underlying model, as illustrated by our analysis of ancient mtDNA in France. Note, however, that a good approximation of the population structure (*Nm*) is important for the efficiency of the spatial test. If the simulated structure is too weak, then the probability of falsely rejecting the null hypothesis of continuity is increased (Table 3). Conversely, a test considering a stronger structure will be too conservative. A careful choice of *Nm* is thus important, either by using information drawn from the literature when it is available, or by investigating a range of values for this parameter. For instance, statistics measuring intra-population diversity such as gene or nucleotide diversity could potentially be used to estimate *Nm* before performing the continuity test. Note that our series of descriptive results obtained in the square world can be qualitatively extrapolated to other kinds of markers, haploid or diploid. Indeed, genetic differentiation between serial samples is expected to be greater under a model including migration and population subdivision than under panmixia, for any kind of molecular markers.

### Applying the spatially explicit continuity test to real data in Europe

We compared the spatial to the non-spatial approaches for testing population continuity with ancient DNA using two real mitochondrial datasets in France and in Germany. Each dataset is composed of an ancient population sample from the early Neolithic period and one modern population sample from approximately the same region. Panmictic models of population continuity lead to sharper distributions of low *Fst* values between the samples considered when compared to equivalent spatial models (Fig. 6). Regarding the German dataset, all four models are concordant with a rejection of the null hypothesis of population continuity (Fig. 6a, b). For the French dataset, both spatially explicit models and one panmictic model (one phase of growth, Fig. 6c) retain the hypothesis of continuity while the other panmictic model rejects continuity at the 5% level (two phases of growth, Fig. 6d). The latter result confirms that the incorporation of population structure and migration in the model used to test for population continuity may significantly affect the results and as a consequence, alter the conclusion. As the spatial dimension undoubtedly mattered during human evolution, we would favour the results obtained with the spatially explicit model over those obtained with the panmictic one. With this additional level of realism, the genetic differentiation of samples is not only a function of demographic variations due to genetic drift but also a function of ongoing local gene flow. Moreover, the *Fst* estimated between the two serial French samples is not significant (*p-value* = 0.08), consistent with the conclusion provided by the spatially explicit continuity test (i.e no rejection of continuity). Furthermore, we noted that the differences between both models are especially big when the ancient sample under study is spatially and temporally homogeneous, like the French one (Additional file 3 Table S2). In the latter situation, using a panmictic model as a basic assumption for testing continuity may be an insufficient approximation, and we would recommend using the spatially explicit approach instead. Note, however, that the choice of the *Nm* value is important as discussed above.

Overall, our results tend to confirm for the maternal line previous studies supporting a rejection of population continuity from early Neolithic to modern times in central Europe [2, 32], potentially explained by an important immigration wave from the East during the Bronze Age [49, 50]. A substantial arrival of immigrant farmers after the early Neolithic phase could also potentially explain this discontinuity. However, there is no evidences for a massive immigration during later Neolithic phases and at contrary, recent studies have suggested that the genetic contribution of local hunter-gatherers has increased with time [36, 51, 52]. Regarding the French dataset, our results challenge the claimed absence of population continuity for the maternal line since the Neolithic period in that area, which has been proposed without a formal test [39]. Retaining the hypothesis of population continuity for the Paris basin may indicate that genetic drift and ongoing gene flow with neighbouring population are sufficient to explain the differences between early Neolithic and modern samples, without the need to invoke an additional genetic input due to an immigration event. Similarly, signal of long-term population continuity (8000 years) for the maternal line has also been found in the South Caucasus [53]. However, retaining the continuity hypothesis could also mean that a genetic shift is not detected either because the test is not powerful enough with the dataset analysed or because there was a genetic input from a population not sufficiently differentiated to let a visible trace in the data. Moreover, because our analysis focuses on one single locus, which is inherited through the maternal line, and because the ancient sample is relatively limited in size and comes from a single archaeological site, it cannot be excluded that future analyses on different loci (Y chromosome, autosomes) or on data from different archaeological sites bring a different answer.

Finding a different result for the French and the German dataset could at a first glance seem conflicting because both areas are separated by less than 1000 km. However, cultural and/or demographic regional heterogeneities between the Paris basin and Eastern France/Western Germany were already detected for the earlier Neolithic period. Altogether, these results call for an independent analysis of each region for which serial ancient genetic material is available. Such a regional and temporal approach has a great potential not only for the study of the Neolithic transition in Europe, but also for studies on more recent historical periods e.g. [6] and other continents e.g. [54].

## Conclusion

We present a new approach to simulate genetic diversity in samples from different times and locations that takes into account population structure, migration and the spatiotemporal variance of lineages within population samples. We show that these factors do have an influence on the genetic relationship measured between serial samples and thus affect the test for population continuity, which is widely applied to aDNA. Our approach is versatile and has the potential to evaluate many different evolutionary scenarios with ancient molecular data. It will thus be particularly useful in analysing new aDNA datasets (uniparental or autosomal) in various research contexts, in humans but also in other species.

## Additional files

Additional file 1: Figure S1. Illustration of the numerical map used for the Panmictic and the spatial scenarios simulated for testing continuity in France and Germany. The Panmictic model is made up of one single deme while the spatial model is made up of approximately 7000 inland demes of 50 *km* × 50 *km*. Maps have been generated with the program SPLATCHE2. (PDF 571 kb)

Additional file 2: Table S1. Parameters for the various continuity scenarios simulated in Europe. (DOCX 18 kb)

Additional file 3: Table S2. Results of the continuity tests applied to datasets from Germany and France under various continuity scenarios. (DOCX 15 kb)
