# Supplementary material for: Investigating population continuity with ancient DNA under a spatially explicit simulation framework
Source: BMC Genet. 2017 Dec 15;18:114. doi: 10.1186/s12863-017-0575-6 (PMC5731203; doi:10.1186/s12863-017-0575-6)
Supplement: Supplementary file 1 — Illustration of the numerical map used for the Panmictic and the spatial scenarios simulated for testing continuity in France and Germany. The Panmictic model is made up of one single deme while the spatial model is made up of approximately 7000 inland demes of 50 km × 50 km. Maps have been generated with the program SPLATCHE2. (PDF 571 kb) [file 12863_2017_575_MOESM1_ESM.pdf]

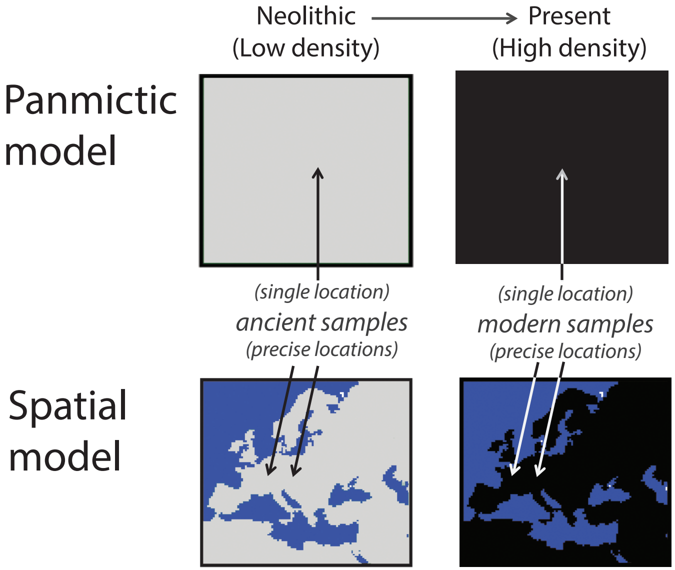
Neolithic
(Low density)
Present
(High density)
Panmictic model
Spatial model
(single location)
ancient samples
(precise locations)
(single location)
modern samples
(precise locations)
