# Supplementary material for: Investigating population continuity with ancient DNA under a spatially explicit simulation framework
Source: BMC Genet. 2017 Dec 15;18:114. doi: 10.1186/s12863-017-0575-6 (PMC5731203; doi:10.1186/s12863-017-0575-6)
Supplement: Supplementary file 2 — Parameters for the various continuity scenarios simulated in Europe. (DOCX 18 kb) [file 12863_2017_575_MOESM2_ESM.docx]

Table S1 Parameters for the various continuity scenarios simulated in Europe

|  | **Panmictic (P)** | | | | | |
| --- | --- | --- | --- | --- | --- | --- |
|  | **10 *ky* population continuity** | | | **40 *ky* population continuity** | | |
| ***Scenarios*** | ***P1-20k*** | ***P1-50k*** | ***P1-100k*** | ***P2-20k*** | ***P2-50k*** | ***P2-100k*** |
| ***Growth Phases*** | 1 | 1 | 1 | 2 | 2 | 2 |
| ***r*** | 0.016 | 0.014 | 0.012 | 0.016 | 0.014 | 0.012 |
| ***t*** | 400 | 400 | 400 | 1,600 | 1,600 | 1,600 |
| ***N_Anc_*** | 20,000 | 50,000 | 100,000 | 5,000 | 5,000 | 5,000 |
| ***N_P_*** | - | - | - | 1,000 | 1,000 | 1,000 |
| ***N_N_*** | 20,000 | 50,000 | 100,000 |  |  |  |
| ***K_P_*** | - | - | - | 20,000 | 50,000 | 100,000 |
| ***K_M_*** | 12,000,000 | 12,000,000 | 12,000,000 | 12,000,000 | 12,000,000 | 12,000,000 |
|  |  |  |  |  |  |  |
|  | **Spatial (SP)** | | | | | |
|  | **10 *ky* population continuity** | | | **40 *ky* population continuity** | | |
| ***Scenarios*** | ***SP1-k100*** | ***SP1-k250*** | ***SP1-k500*** | ***SP2-k100*** | ***SP2-k250*** | ***SP2-k500*** |
| ***Growth Phases*** | 1 | 1 | 1 | 2 | 2 | 2 |
| ***r*** | 0.8 | 0.8 | 0.8 | 0.4 | 0.4 | 0.4 |
| ***t*** | 400 | 400 | 400 | 1,600 | 1,600 | 1,600 |
| ***m*** | 0.25 | 0.25 | 0.25 | 0.25 | 0.25 | 0.25 |
| ***N_Anc_*** | 20,000 | 50,000 | 100,000 | 5,000 | 5,000 | 5,000 |
| ***N_P_*** | - | - | - | 1,000 | 1,000 | 1,000 |
| ***N_N_*** | 20,000 | 50,000 | 100,000 | - | - | - |
| ***K_P_*** | - | - | - | 40 | 40 | 40 |
| ***K_N_*** | 100 | 250 | 500 | 100 | 250 | 500 |
| ***K_M_*** | 24,000 | 24,000 | 24,000 | 24,000 | 24,000 | 24,000 |
| ***Nm_N_*** | 25 | 62.5 | 125 | 25 | 62.5 | 125 |

All parameters are those implemented in SPLATCHE2 [1]. r = growth rate, t = number of generations simulated, N_anc_= ancestral size, N_UP_ = initial Paleolithic population size, N_N_ = initial Neolithic population size, m=migration rate, K_P_ = Paleolithic carrying capacity, K_N_= Neolithic carrying capacity, K_M_= Modern time carrying capacity. All population sizes are given in number of effective genes.

*N_anc_* represents the population size in Europe during the Palaeolithic before the onset of the Neolithic increase for the 10ky continuity models and it represents the population size in Africa before the onset of the Palaeolithic increase in Europe for 40ky continuity models. When *N_anc_* > *N_UP_*, it represents a population bottleneck occurring at the onset of the European colonization by modern humans.

1. Ray N, Currat M, Foll M, Excoffier L: **SPLATCHE2: a spatially explicit simulation framework for complex demography, genetic admixture and recombination**. *Bioinformatics* 2010, **26**(23):2993-2994.
