# Supplementary material for: Investigating population continuity with ancient DNA under a spatially explicit simulation framework
Source: BMC Genet. 2017 Dec 15;18:114. doi: 10.1186/s12863-017-0575-6 (PMC5731203; doi:10.1186/s12863-017-0575-6)
Supplement: Supplementary file 3 — Results of the continuity tests applied to datasets from Germany and France under various continuity scenarios. (DOCX 15 kb) [file 12863_2017_575_MOESM3_ESM.docx]

Table S2 Results of the continuity tests applied to datasets from Germany and France under various scenarios

|  |  | *1 phase of growth* | | | *2 phases of growth* | | | |
| --- | --- | --- | --- | --- | --- | --- | --- | --- |
|  |  | **Panmictic (P)** | | | | | | |
|  |  | **10 *ky* population continuity** | | | | **40 *ky* population continuity** | | |
|  | *Scenarios* | *P1-20k* | *P1-50k* | *P1-100k* | | *P2-20k* | *P2-50k* | *P2-100k* |
| ***Germany*** | *P_sim>obs_* | **0.030** | **0.011** | **< 0.001** | | **< 0.001** | **< 0.001** | **0.001** |
|  | *Bonferroni* | 0.090 | **0.033** | **< 0.001** | | **< 0.001** | **< 0.001** | **0.003** |
| ***France*** | *P_sim>obs_* | 0.197 | 0.060 | **0.010** | | **0.047** | **0.010** | **0.040** |
|  | *Bonferroni* | 0.660 | 0.180 | **0.030** | | 0.120 | **0.030** | 0.120 |
|  |  | **Spatial (SP)** | | | | | | |
|  |  | **10 *ky* population continuity** | | | | **40 *ky* population continuity** | | |
|  | *Scenarios* | *SP1-k100* | *SP1-k250* | *SP1-k500* | | *SP2-k100* | *SP2-k250* | *SP2-k500* |
| ***Germany*** | *P_sim>obs_* | **0.049** | **0.032** | **0.033** | | **0.004** | **0.002** | **0.001** |
|  | *Bonferroni* | 0.147 | 0.096 | 0.099 | | **0.012** | **0.006** | **0.003** |
| ***France*** | *P_sim>obs_* | 0.510 | 0.429 | 0.290 | | 0.911 | 0.576 | 0.304 |
|  | *Bonferroni* | 1.000 | 1.000 | 0.870 | | 1.000 | 1.000 | 0.912 |

The proportion of simulations resulting in a *Fst* between modern and ancient samples bigger than the observation (*P_sim_*_>_*_obs_*) is given for all tested continuity scenarios. Values after Bonferonni correction for multiple tests are also given for each model family. Significant values at the 5% level are bold. Gray background indicates the most comparable scenario among model families using the most realistic parameter values given prior information (see main text).
